# Drug-Coated Balloons versus Drug-Eluting Stents for the Treatment of De Novo Coronary Artery Disease: A Meta-Analysis of Randomized Controlled Trials

**DOI:** 10.31083/j.rcm2512446

**Published:** 2024-12-19

**Authors:** Jialong Niu, Kexin Wang, Wenjie Wang, Yixuan Liu, Jiaxin Yang, Yan Sun, Furong Wang, Wen Gao, Hailong Ge

**Affiliations:** ^1^Department of Cardiology, Beijing Anzhen Hospital, Capital Medical University, 100069 Beijing, China; ^2^Department of Cardiology, Inner Mongolia Ordos City Dalate Banner People’s Hospital, 017000 Ordos, Inner Mongolia Autonomous Region, China; ^3^Department of Cardiology, Bayannaoer City Hospital, 015000 Bayannaoer, Inner Mongolia Autonomous Region, China

**Keywords:** drug-coated balloons, drug-eluting stents, de novo coronary artery disease, meta-analysis, randomized controlled trials

## Abstract

**Background:**

Because of the limitations in new-generation drug-eluting stents (DES), treatments advocating for non-stents with a drug-coated balloon (DCB) is now of great interest. Here, we conducted a meta-analysis to testify whether a DCB was more effective and safer than a DES in treating de novo coronary artery disease (CAD).

**Methods:**

We searched PubMed, Embase, Cochrane Library, and Web of Science to obtain high-quality trials comparing DCB with DES for the treatment of de novo CAD. The primary endpoint was target lesion revascularization (TLR), and the secondary endpoints were in-lesion late lumen loss (LLL), all-cause death, myocardial infarction and binary restenosis.

**Results:**

We enrolled 1661 patients from seven randomized clinical trials. Compared with the DES group, the MD (mean difference) of in-lesion LLL was significantly lower in the DCB group (MD –0.19, 95% CI –0.23 to –0.16, *p* < 0.00001, I^2^ = 0%). The DCB group showed superiority in small vessel disease (SVD) in in-lesion LLL (MD –0.21, 95% CI –0.34 to –0.08, *p* = 0.001).

**Conclusions:**

The DCB group exhibited a lower in-lesion LLL compared to the DES group, and DCB was not inferior to DES in other endpoints, including in the SVD subgroup. Hence, to our knowledge, DCB is non-inferior to DES for de novo CVD and SVD. DCB in patients with CVD needs further large and long-term clinical trials to demonstrate its long-term efficacy.

**The PROSPERO Registration:**

CRD42021268965, https://www.crd.york.ac.uk/prospero/display_record.php?RecordID=268965.

## 1. Introduction

With rapid advances in technology, most patients with coronary artery disease (CAD) needing percutaneous coronary intervention (PCI) are now treated with stent implantation [1]. Compared with bare-metal stents (BMS), the new-generation drug-eluting stents (DES) have significantly reduced the rate of restenosis and late lumen loss (LLL) in long-term follow-up after PCI. However, DES is not effective and safe enough in treating certain complex coronary artery lesions, such as bifurcation lesions where complete coverage of the lesion may not be achievable, or in cases where there is stent strut overlap during treatment [2] and small vessel disease (SVD), in which the diameter of the target vessel is less than 2.75 mm where stent implantation is not feasible [3]. Some patients also exhibit psychological resistance to having stents implanted in their bodies and prefer treatment without implantable devices. Thus, based on the limitations of DES mentioned above, the treatment advocating non-stent based on local drug delivery with a drug-coated balloon (DCB) has emerged as a new strategy during recent years [4].

A DCB is a balloon coated with a high dose of pharmacologically active substances, which can transfer anti-proliferative drugs (e.g., paclitaxel, zotarolimus and sirolimus) into the specific vessel walls during a 30–60 seconds single inflation without implants in the coronary artery [5, 6]. But previously published research has been more concentrated on treating in-stent restenosis (ISR) and less focused on de novo coronary artery disease. Recently, several randomized controlled trials (RCTs) have shown that DCB has a profit in terms of LLL in de novo CAD [3, 7, 8, 9, 10]. However, there are studies also confirming that DCB was non-superior to DES in the endpoints of clinical efficacy and safety, even more disadvantaged in reducing the need for target lesion revascularization (TLR) at 3 years in de novo CAD [11], for only the delivery of drugs on the balloon were not sufficient enough to produce a permanent and homogeneous effect on the arterial wall [12]. That also explains why DCB in de novo CAD is not the first-line treatment recommended by the revascularization guidelines currently [13].

Therefore, we conducted a collaborative meta-analysis to summarize the evidence from RCTs that compared long-term outcomes between DCB and DES, to testify whether DCB was more effective and safer than DES in treating de novo coronary artery disease.

## 2. Materials and Methods

### 2.1 Study Design and Search Strategy

The study was an investigator-initiated, collaborative meta-analysis of individual patient data published in RCTs. Studies that met the following eligibility criteria would be included: (1) randomized clinical trials; (2) published in English language; (3) patients with de novo CAD; (4) compared DCB to DES; (5) clinical follow-up of at least 6 months; (6) reported with complete and accurate data. Sub-studies of large studies were excluded.

Multiple electronic databases (PubMed, Embase, Cochrane Library, and Web of Science) were searched from 1 August 2010 to 1 August 2023 in order to obtain high-quality research. The search terms include: “Drug-Coated Balloons”, “DCB”, “Drug-Eluting Stents”, “DES”, “De novo coronary disease”, “De novo coronary lesions”, “Coronary Artery Disease”, “Randomized Controlled Trials” and “RCT”. The full search strategy for each database is present in **Supplementary material 1**. All studies were screened through titles and abstracts, then reviewed in full text to determine if they met the inclusion criteria.

This study was designed and conducted according to the Preferred Reporting Items for Systematic Reviews and Meta-Analyses (PRISMA) guidelines [14]. The protocol was registered with PROSPERO (CRD42021268965). Furthermore, all selection processes, data extraction and statistical analyses were conducted by two independent investigators, respectively. And if there was any disagreement, a third person would be involved to negotiate and make the final decision.

### 2.2 Endpoint

The primary endpoint was TLR, defined as any revascularization at the target segment [15]. The secondary endpoints were in-lesion LLL, all-cause death, myocardial infarction (MI) and binary restenosis (BR). LLL was calculated as the difference in the value of in-stent minimal lumen diameter (MLD) at the target segment between the immediate postoperative period and during the follow-up [16]. Death and MI were defined according to the guideline of the Academic Research Consortium [15]. BR was defined as diameter stenosis >50% at the target segment during the follow-up angiogram [17]. In addition, the surgery-related indicators such as TLR, LLL and BR were considered as efficacy outcomes, while the clinical outcomes, such as all-cause death and MI were considered as safety outcomes.

### 2.3 Statistical Analysis

The data were analysed by Review Manager 5.3 ( The Nordic Cochrane Centre, The Cochrane Collaboration, Copenhagen, Denmark​) and Stata 14.0 (StataCorp LLC, College Station, TX, USA​) software. All potential sources of bias were assessed by using the Cochrane Collaboration tool [18]. In each included trial, categorical variables were reported as absolute numbers and continuous variables were reported as the mean ± standard deviation (SD). Risk ratio (RR), MD and their 95% confidence intervals (CI) were used as the effect indicators (*p* < 0.05 was considered statistically significant). The heterogeneity of the included trials was assessed by Cochrane’s Q and I^2^ [19]. And the values of I^2^ <25%, between 25% and 50%, and >50% were described as low, intermediate, or severe heterogeneity, respectively [20]. If both *p* > 0.10 and I^2^ <50% were satisfied, a fixed-effects model and Mantel-Haenszel method was conducted to combine RRs [21, 22]. The inverse variance method was used to analyse continuous variables [23]. In addition, subgroup analyses were performed according to the diameter of target vessels. Finally, we conducted the sensitivity analysis and publication bias test, which was explored by the funnel plots and Egger test (*p* < 0.05 was considered statistically significant).

## 3. Results

### 3.1 Study Characteristics and Quality Assessment

**Fig. 1.**
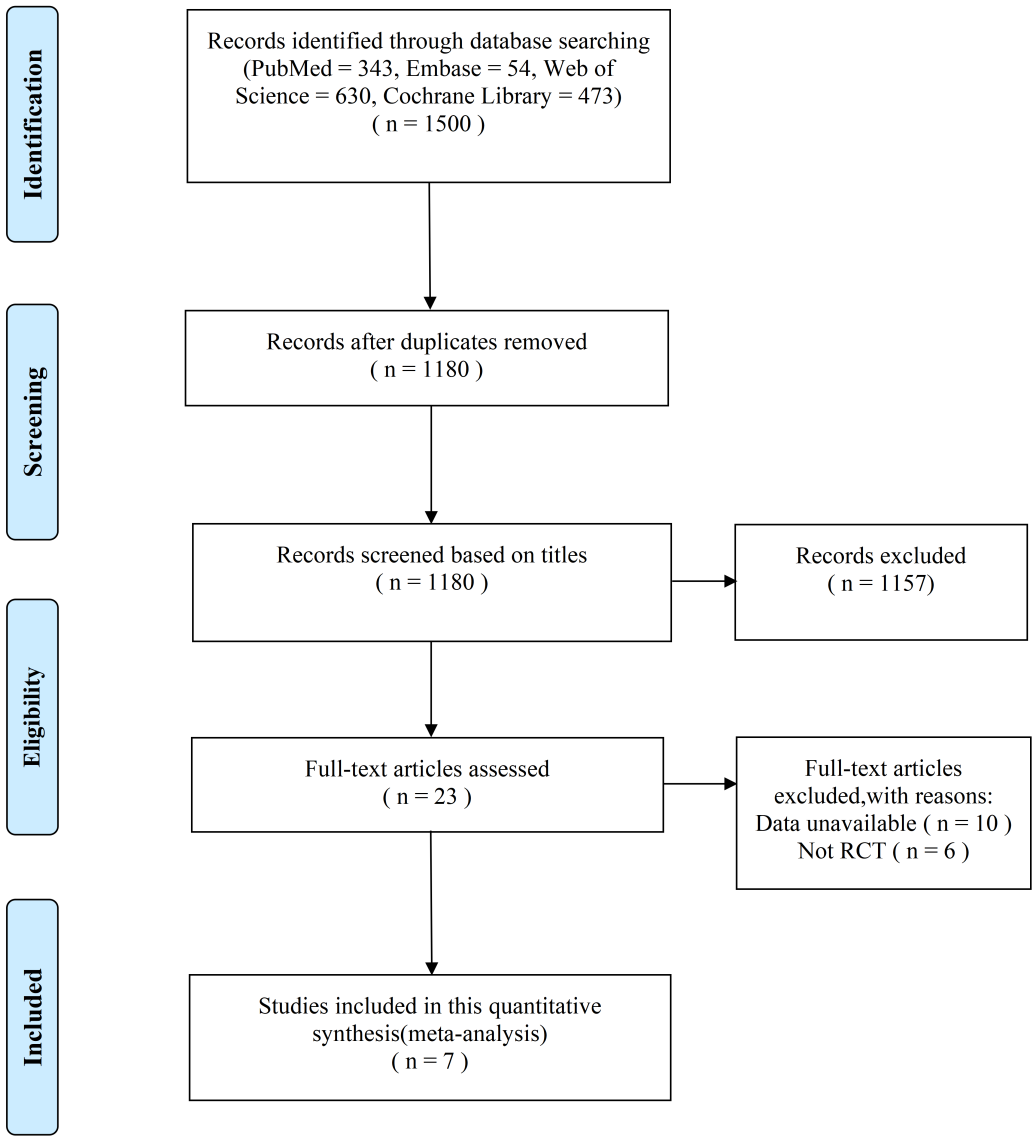
**Summary of the study selection process**. The stages of the study selection process are highlighted with the number of studies selected and the number of studies excluded at each stage. RCT, randomized controlled trials.

The initial search criteria showed 1661 results. After removing duplicates, screening through abstracts and reviewing 31 full-text articles, 7 studies were finally included in this meta-analysis. Details about the selecting processes were shown in Fig. 1. A total of 1661 patients were included, with 838 assigned to the DCB group and 823 assigned to the DES group. We extracted the number of patients in each group, the types of devices, clinical data, follow-up time, endpoints and other information from the included trials. Baseline characteristics of these trials were summarized in Table 1 (Ref. [3, 7, 8, 9, 11, 24, 25]) and **Supplementary material 2**. Among these research, patients from 5 trials had SVD [3, 8, 11, 24, 25]. The follow-up time of 2 trials were 6 months [3, 7], 1 trial were 9 months [24], and 4 trials were more than a year [8, 9, 11, 25].

**Table 1.** **Main characteristics of the included randomized clinical trials**.

Study	Disease	Number of patients		Type of devices		Follow-up time (mts)
		DCB	DES	DCB	DES	
Latib 2012 BELLO [8]	Small Coronary Vessel Disease	90	92	IN.PACT Falcon DCB	Taxus Libertè DES	6 (angio), 6–36 (clinical)
Cortese 2010 PICCOLETO [24]	Small Coronary Vessel Disease	29	31	the Dior PCB	Taxus Libertè DES	9 (angio), 8 (clinical)
Tang 2018 RESTORE SVD China [11]	Small Coronary Vessel Disease	116	114	Restore DCB	RESOLUTE DES	up to 60
Cortese 2020 PICCOLETO II [3]	Small Coronary Vessel Disease	118	114	Elutax SV DCB	Xience Prime DES	6
Jeger 2020 BASKET-SMALL 2 [25]	Small Coronary Vessel Disease	382	376	SeQuent Please DCB	Xience Prime, Taxus Element DES	36
Yu 2021 [9]	De novo coronary disease	85	85	SeQuent Please DCB	Xience Prime DES	9 (angio), 12 (clinical)
Gobić 2017 [7]	De novo coronary disease	41	37	SeQuent Please DCB	Biomime DES	6

DCB, drug-coated balloon; DES, drug-eluting stent; mts, months; angio, follow-up with coronary angiographic outcomes; clinical, follow-up with clinical outcomes; SV, small vessel.

**Fig. 2.**
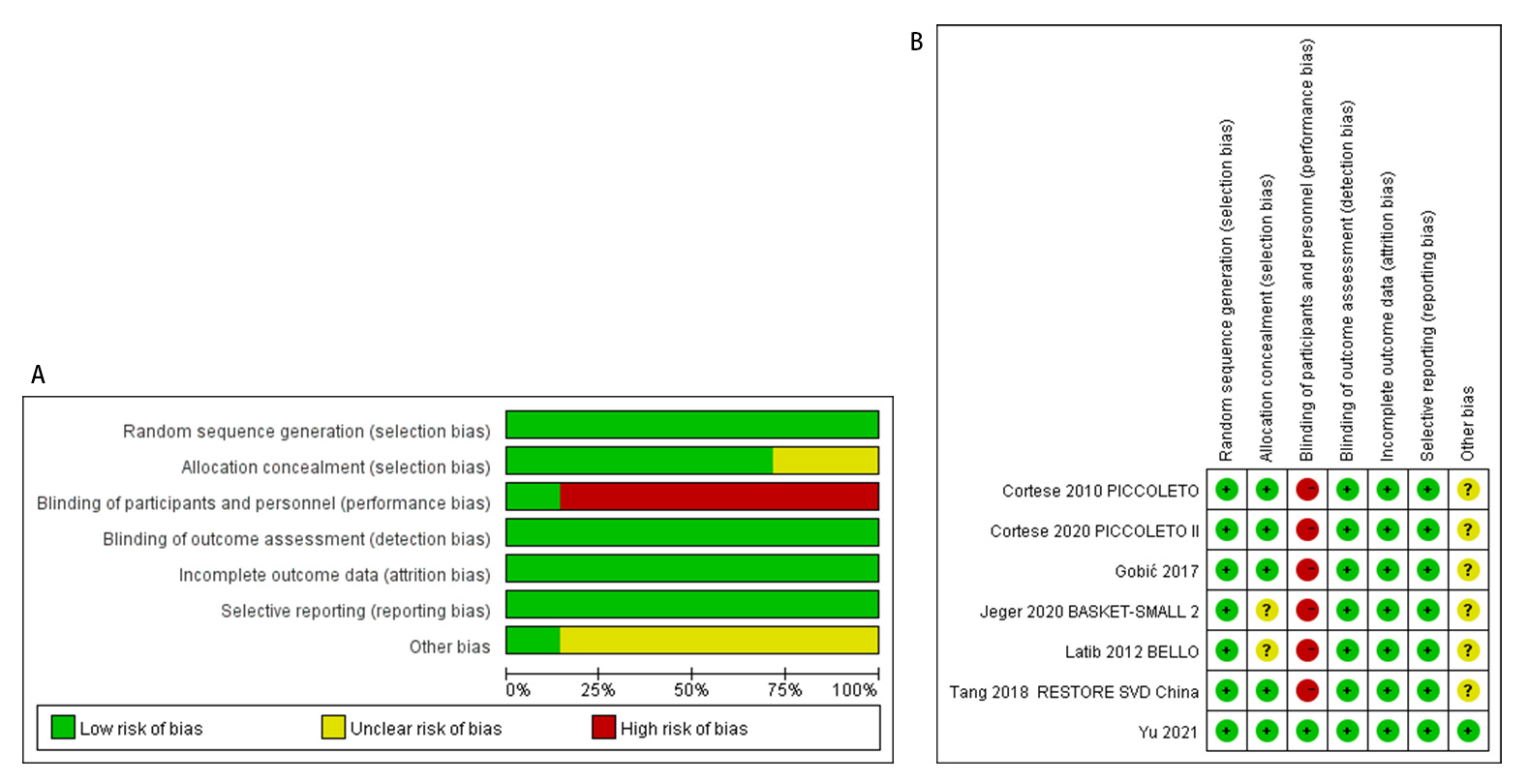
**Risk of bias assessment**. (A) Risk of bias graph. (B) Risk of bias summary.

According to the Cochrane Collaboration’s tool, the quality assessment of included studies was summarized in Fig. 2. All the studies followed the principle of random allocation. 5 studies showed the method of allocation concealment, such as using a randomly permuted blocks method [3, 7, 9, 11, 24]. 1 study was double-blind [9]. And there were no evidence of significant detection bias, attrition bias and reporting bias. Furthermore, except for 1 study [9], bias from other sources was unclear.

### 3.2 Endpoint

#### 3.2.1 The Primary Endpoint

**Fig. 3.**
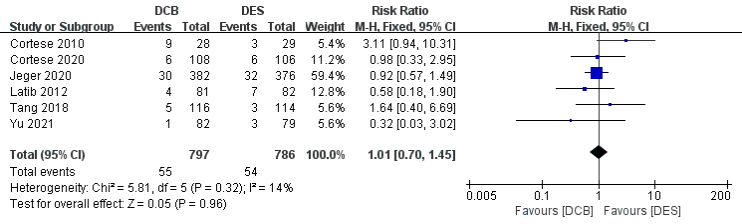
**Forest plot of target lesion revascularization (TLR)**. DCB, drug-coated balloon; DES, drug-eluting stent; M-H, Mantel-Haenszel method.

Due to the low heterogeneity (*p* > 0.10 and I^2^ <50%), a fixed-effects model was adopted. As the forest plots showed in Fig. 3, 7 randomized trials and a total of 1661 patients were involved [3, 7, 8, 9, 11, 24, 25]. And the DCB group is non-inferior to the DES group in preventing TLR (RR 1.01, 95% CI 0.7 to 1.45, *p* = 0.96). Jeger *et al*. [25] had the highest weight (59.4%).

#### 3.2.2 The Secondary Endpoint

**Fig. 4.**
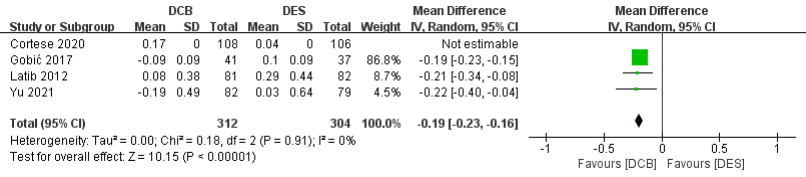
**Forest plot of in-lesion late lumen loss (LLL)**. DCB, drug-coated balloon; DES, drug-eluting stent; IV, inverse variance.

**Fig. 5.**
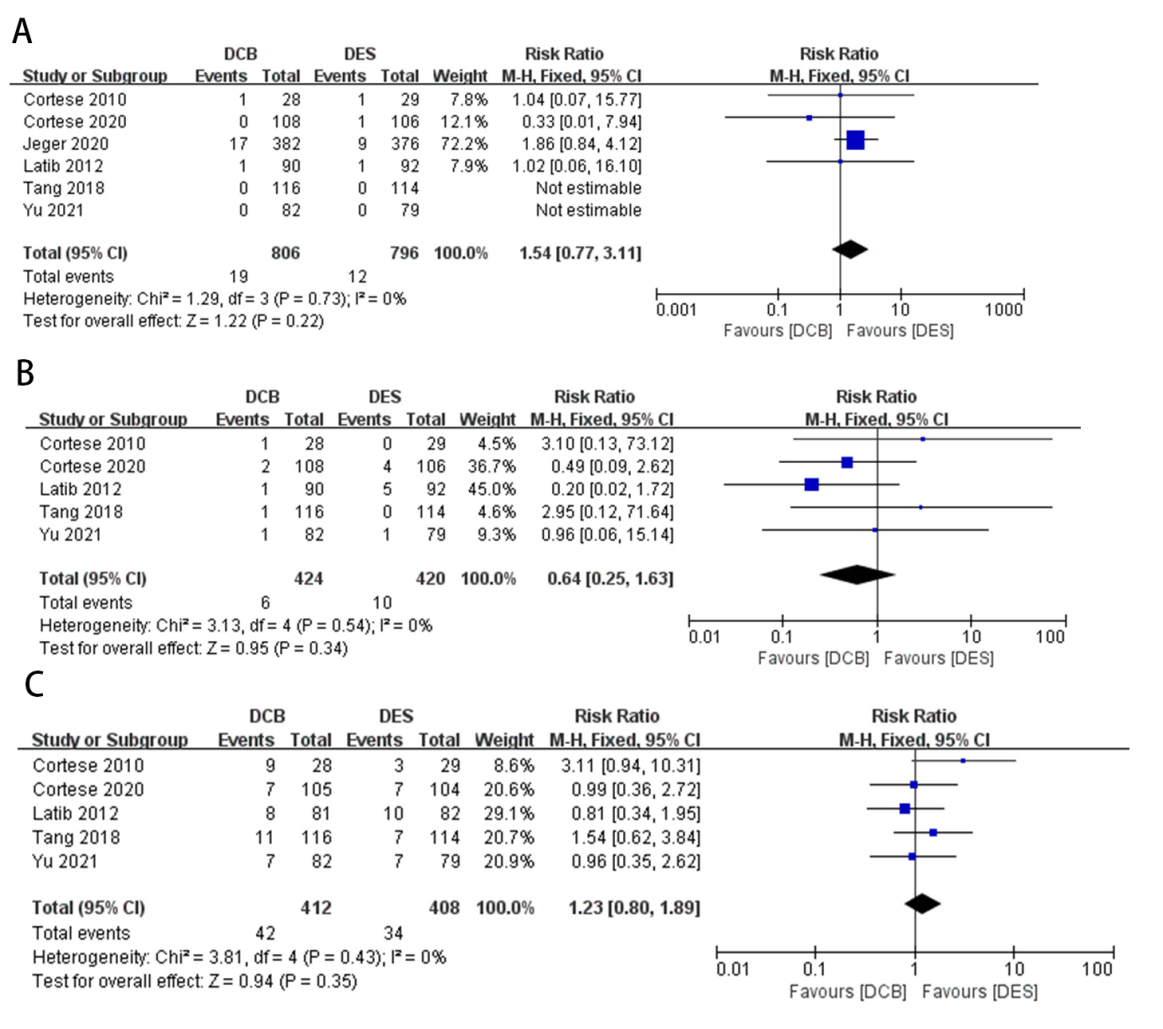
**Forest plot of other endpoints**. (A) All-cause death. (B) Myocardial infarction (MI). (C) Binary restenosis (BR). DCB, drug-coated balloon; DES, drug-eluting stent; M-H, Mantel-Haenszel method.

We used a fixed-effects model in in-lesion LLL, all-cause death, MI and BR (with all *p* > 0.10 and I^2^ <50%). 4 studies recorded in-lesion LLL, and Gobić [7] had the highest weight (86.8%) [3, 7, 8, 9]. 6 studies recorded all-cause death, and Jeger *et al*. [25] had the highest weight (72.2%) [3, 8, 9, 11, 24, 25]. 5 studies recorded MI, and Latib *et al*. [8] had the highest weight (45.0%) [3, 8, 9, 11, 24]. 5 studies recorded BR, and Labit *et al*. [8] had the highest weight (29.1%) [3, 8, 9, 11, 24]. Compared with the DES group, the combined MD of in-lesion LLL was significantly lower in the DCB group (MD –0.19, 95% CI –0.23 to –0.16, *p* < 0.00001, I^2^ = 0%). And Jeger *et al*. [25] had the highest weight (46.2%). Our analysis showed there were no significant differences between the DCB group and the DES group in all-cause death (RR 1.54, 95% CI 0.77 to 3.11, *p* = 0.22), MI (RR 0.64, 95% CI 0.25 to 1.63, *p* = 0.34) and BR (RR 1.23, 95% CI 0.80 to 1.89, *p* = 0.35). The forest plots of in-lesion LLL, all-cause death, MI, and BR were shown in Figs. 4,5.

### 3.3 Subgroup Analysis

To better compare the differences between DES and DCB, we further conducted a subgroup analysis on SVD, the rusults are present in **Supplementary material 3**. Compared with the DES group, the DCB group also showed superiority in SVD patients in in-lesion LLL (MD –0.21, 95% CI –0.34 to –0.08, *p* = 0.001). No notable difference in TLR (RR 1.05, 95% CI 0.73 to 1.52, *p* = 0.79), all-cause death, MI and BR were observed in other endpoints.

### 3.4 Sensitivity Analysis and Publication Bias Test

**Fig. 6.**
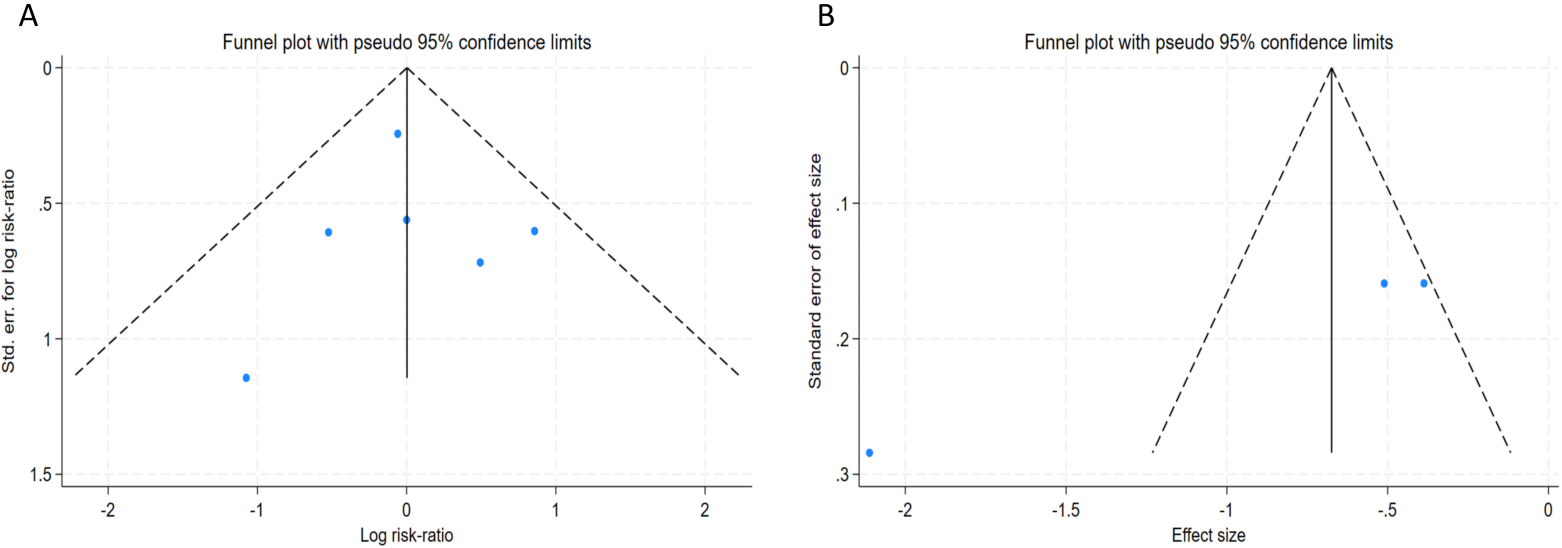
**Funnel plot of publication bias**. (A) Target lesion revascularization (TLR). (B) In-lesion late lumen loss (LLL).

After removing the included trials one by one, in addition to in-lesion LLL, the results did not show a significant difference when the RR of other endpoints were recombining together. Compared with the DES group, the results after removing Gobić *et al*. [7] showed an increase in the in-lesion LLL of the DCB group, while removing Yu *et al*. [9] resulted in a decrease. Nevertheless, both results support the advantage of DCB in in-lesion LLL. More figures were shown in **Supplementary material 4**. We tested the publication bias by constructing funnel plots (Fig. 6) and performed the Egger test. According to the TLR (*p* = 0.8807), no significant publication bias was found. However, there was a notable publication bias in the in-lesion LLL (*p* < 0.0001).

## 4. Discussion

In this large collaborative meta-analysis of 7 RCTs, we enrolled a total of 1661 patients undergoing treatment for de novo CAD to assess the clinical efficacy and safety of DCB and DES. To the best of our knowledge, the main findings of this paper are as follows:

(1) Compared with the DES group, the DCB group had a lower risk of in-lesion LLL, and DCB did not show significant inferiority to DES in other endpoints.

(2) In the subgroup of SVD, the use of DCB was linked to reduced risks of in-lesion LLL in patients with SVD. The rates of all-cause death and MI were found similar in both groups.

To reduce the incidence of malignant events (e.g., cardiac death, stroke and MI) after PCI, a series of new-generation DES gradually replaced bare-metal stents in the first line of coronary intervention treatments, with the development of stent materials and eluting-drugs [13]. Compared with BMS and first-generation DES, the second-generation DES has significantly improved clinical safety and efficacy outcomes [26]. But there were still plenty of unsolved problems after stent implantation, such as in-stent thrombosis, delayed healing, local hypersensitivity reactions and neo-atherosclerosis, which could lead to a steady increase in major adverse cardiovascular events (MACEs) [27]. To overcome the limitations mentioned above, more and more medical professionals suggested PCI without stent implantation, which has pursued the development of DCB [5, 12]. DCB is conventional semi-compliant angioplasty balloon coated with a high dose of pharmacologically active substances, which can transfer anti-proliferative drugs into the target arterial wall. This treatment not only alleviates stenosis but also inhibits cellular proliferation at the target arterial wall, thereby reducing the incidence of restenosis. Compared to DES, the DCB do not require a permanent implant and necessitate a shorter duration of dual antiplatelet therapy post-procedure. However, DCB lacks the long-term structural support provided by DES [5, 6, 17].

Nowadays, DES is predominantly used in clinical settings, and several studies have been conducted to compare DCB with DES in clinical effectiveness and safety of de novo CAD. The results of Gobić *et al*. [7] confirmed that DCB showed favorable clinical and angiographic outcomes in 6 months follow-up in patients with de novo CAD. Similarly, in patients with small vessel disease, the BASKET-SMALL 2 study and the BELLO (Balloon Elution and Late Loss Optimization) study showed a preference to DCB and demonstrated that it was non-inferior to DES in the rates of restenosis and revascularization [8, 25]. However, the PICCOLETO (Drug Eluting Balloon Efficacy for Small Coronary Vessel Disease Treatment) trial came to the opposite conclusion [24]. The previous meta-analysis put forward that compared with DES, DCB showed similar clinical efficacy and safety outcomes [28, 29, 30]. Therefore, DCB could be considered as an alternative choice in the clinical treatment of CAD. In this research, we summarized the evidence from all available RCTs about de novo CAD before. As our results showed, there was no significant difference presenting in TLR, all-cause death, MI and BR between DCB and DES in the follow-up, which was the same as the studies mentioned above. Additionally, we also found that the DCB group had more potential in reducing in-lesion LLL.

Small vessel disease is associated with an increased risk of restenosis and stent thrombosis following the deployment of DES. Accordingly, the strategy of treating SVD through the direct administration of an antirestenotic DCB, thereby avoiding the implantation of stents, has garnered significant interest. Furthermore, we also conducted a subgroup analysis comparing DCB versus DES in patients with SVD. In patients with SVD, it seems that DCB was more effective in reducing the occurrence of in-lesion LLL, and there were no differences in other outcomes.

Limitation: Even if our large, comprehensive meta-analysis enrolled more than 1600 patients, there were still some limitations associated with the original trials that cannot be ignored. First, the different types of DCB or DES used in the 7 included trials may cause heterogeneity in our analysis. The studies we included had both the first and the second-generation DES. Compared with the second-generation DES, the first generation DES had a considerable risk of in-stent thrombosis, especially for late (less than 1 year after PCI) and very late stent thrombosis (more than 1 year after PCI) [1]. Thus, we also conducted a sensitivity analysis, in which the results did not show a significant difference except for in-lesion LLL. In addition, the current studies predominantly focus on first- or second-generation DES and paclitaxel-coated balloons. However, the technique has evolved, and sirolimus-coated balloons are now available as well. Our study did not conduct a subgroup analysis of these various DCBs due to insufficient data. Second, although publication bias does exist in the research, it only affects our evaluation of the efficacy of DCB in reducing LLL. This bias does not alter the demonstrated superiority of DCB over DES. Consequently, even if our assessment of the specific numerical reduction in LLL by DCB might not be entirely accurate, the overall advantage of DCB compared to DES remains valid. And there were only two available RCTs that reported the figure of in-lesion LLL for DCB versus DES in SVD. So our results in in-lesion LLL of DCB for patients with SVD should be interpreted with caution. Finally, although we enrolled a large de novo CAD patient population, we have not got specific individual patient data. Thus, we could not design more subgroup analyses about other meaningful clinical statuses (e.g., diabetes, STEMI).

DCB has been reported in the majority of clinical trials and meta-analyses in treating ISR for its advantageous clinical outcomes. Different from the present studies, our study was concentrated on DCB versus DES in treating de novo CAD rather than ISR. Because of the limitations in new-generation DES, the treatment advocating non-stent based on DCB is now of great interest. In follow-up periods predominantly within one year, the current evidence supports that DCB was not inferior to DES in the clinical efficacy and safety of treating de novo CAD. Therefore, more RCTs are needed in the future to prove which type of treatment could provide more late advantages over hard end-points in patients with de novo CAD.

## 5. Conclusions

In this meta-analysis, we found that the DCB group had more potential in reducing in-lesion LLL and the incidence of TLR, but all-cause death MI and BR were similar between DES and DCB alone, this was also true in SVD. Hence, to our knowledge, DCB is non-inferior to DES in terms of short-term efficacy for de novo CVD and SVD. DCB in patients with CVD needs further large and long-term clinical trials to demonstrate its long-term efficacy.

## Data Availability

The datasets used and/or analyzed during the current study are available from the corresponding author on reasonable request.

## Abbreviations

CAD, coronary artery disease; DCB, drug-coated balloons; DES, drug-eluting stents; BMS, bare-mental stent; PCI, percutaneous coronary intervention; ISR, in-stents restenosis; MACEs, major adverse cardiovascular events; TLR, target lesion revascularization; in-lesion LLL, in-lesion late lumen loss; MI, myocardial infarction; BR, binary restenosis; MLD, minimal lumen diameter; DM, diabetes mellitus; SVD, small vessel disease; CABG, coronary artery bypass grafting; M-H, Mantel-Haenszel method.

## Supplementary Material

Supplementary material associated with this article can be found, in the online version, at https://doi.org/10.31083/j.rcm2512446.
