# Supplementary material for: Drug-Coated Balloons versus Drug-Eluting Stents for the Treatment of De Novo Coronary Artery Disease: A Meta-Analysis of Randomized Controlled Trials
Source: Rev Cardiovasc Med. 2024 Dec 19;25(12):446. doi: 10.31083/j.rcm2512446 (PMC11683689; doi:10.31083/j.rcm2512446)
Supplement: Supplementary file 1 [file 2153-8174-25-12-446-s1.zip › Supplemental material 1.docx]

PubMed:

(((((((((((((((Drug Eluting Stents[Title/Abstract]) OR (Stents, Drug-Eluting[Title/Abstract])) OR (Stents, Drug Eluting[Title/Abstract])) OR (Drug-Eluting Stent[Title/Abstract])) OR (Drug Eluting Stent[Title/Abstract])) OR (Stent, Drug-Eluting[Title/Abstract])) OR (Drug-Coated Stents[Title/Abstract])) OR (Drug Coated Stents[Title/Abstract])) OR (Stents, Drug-Coated[Title/Abstract])) OR (Stents, Drug Coated[Title/Abstract])) OR (Drug-Coated Stent[Title/Abstract])) OR (Drug Coated Stent[Title/Abstract])) OR (Stent, Drug-Coated[Title/Abstract])) AND (((((((((((((Drug Eluting Balloons[Title/Abstract]) OR (Balloons, Drug-Eluting[Title/Abstract])) OR (Balloons, Drug Eluting[Title/Abstract])) OR (Drug-Eluting Balloon[Title/Abstract])) OR (Drug Eluting Balloon[Title/Abstract])) OR (Balloon, Drug-Eluting[Title/Abstract])) OR (Drug-Coated Balloons[Title/Abstract])) OR (Drug Coated Balloons[Title/Abstract])) OR (Balloons, Drug-Coated[Title/Abstract])) OR (Balloons, Drug Coated[Title/Abstract])) OR (Drug-Coated Balloon[Title/Abstract])) OR (Drug Coated Balloon[Title/Abstract])) OR (Balloon, Drug-Coated[Title/Abstract]))) AND ((((((((((De novo coronary lesions[Title/Abstract]) OR (De novo coronary disease[Title/Abstract])) OR (de novo coronary stenosis[Title/Abstract])) OR (de novo small coronary artery disease[Title/Abstract])) OR (de novo small coronary artery lesions[Title/Abstract])) OR (de novo coronary bifurcation lesions[Title/Abstract])) OR (de novo coronary bifurcation disease[Title/Abstract])) OR (Coronary Artery Disease[Title/Abstract])) OR (Artery Disease, Coronary[Title/Abstract])) OR (Artery Diseases, Coronary[Title/Abstract]))) AND ((((randomized controlled trial[Title/Abstract]) OR (randomized[Title/Abstract])) OR (placebo[Title/Abstract])) OR (RCT[Title/Abstract]))

Embase:

#1

'drug eluting stents':ab,ti OR 'stents, drug-eluting':ab,ti OR 'stents, drug eluting':ab,ti OR 'drug-eluting stent':ab,ti OR 'drug eluting stent':ab,ti OR 'stent, drug-eluting':ab,ti OR 'drug-coated stents':ab,ti OR 'drug coated stents':ab,ti OR 'stents, drug-coated':ab,ti OR 'stents, drug coated':ab,ti OR 'drug-coated stent':ab,ti OR 'drug coated stent':ab,ti OR 'stent, drug-coated':ab,ti

#2

'drug eluting balloons':ab,ti OR 'balloons, drug-eluting':ab,ti OR 'balloons, drug eluting':ab,ti OR 'drug-eluting balloon':ab,ti OR 'drug eluting balloon':ab,ti OR 'balloon, drug-eluting':ab,ti OR 'drug-coated balloons':ab,ti OR 'drug coated balloons':ab,ti OR 'balloons, drug-coated':ab,ti OR 'balloons, drug coated':ab,ti OR 'drug-coated balloon':ab,ti OR 'drug coated balloon':ab,ti OR 'balloon, drug-coated':ab,ti

#3

'de novo coronary lesions':ab,ti OR 'de novo coronary disease':ab,ti OR 'de novo coronary stenosis':ab,ti OR 'de novo small coronary artery disease':ab,ti OR 'de novo small coronary artery lesions':ab,ti OR 'de novo coronary bifurcation lesions':ab,ti OR 'de novo coronary bifurcation disease':ab,ti OR 'coronary artery disease':ab,ti OR 'artery disease, coronary':ab,ti OR 'artery diseases, coronary':ab,ti

#4

'randomized controlled trial':ab,ti OR 'randomized':ab,ti OR 'placebo':ab,ti OR 'rct':ab,ti

#5

#1 AND #2 AND #3 AND #4

Web of Science

#1

TS=(Drug Eluting Stents OR Stents, Drug-Eluting OR Stents, Drug Eluting OR Drug-Eluting Stent OR Drug Eluting Stent OR Stent, Drug-Eluting OR Drug-Coated Stents OR Drug Coated Stents OR Stents, Drug-Coated OR Stents, Drug Coated OR Drug-Coated Stent OR Drug Coated Stent OR Stent, Drug-Coated)

#2

TS=(Drug Eluting Balloons OR Balloons, Drug-Eluting OR Balloons, Drug Eluting OR Drug-Eluting Balloon OR Drug Eluting Balloon OR Balloon, Drug-Eluting OR Drug-Coated Balloons OR Drug Coated Balloons OR Balloons, Drug-Coated OR Balloons, Drug Coated OR Drug-Coated Balloon OR Drug Coated Balloon OR Balloon, Drug-Coated)

#3

TS=(De novo coronary lesions OR De novo coronary disease OR de novo coronary stenosis OR de novo small coronary artery disease OR de novo small coronary artery lesions OR de novo coronary bifurcation lesions OR de novo coronary bifurcation disease OR Coronary Artery Disease OR Artery Disease, Coronary OR Artery Diseases, Coronary)

#4

TS=(randomized controlled trial OR randomized OR placebo OR RCT)

#5

#1 AND #2 AND #3 AND #4

Cochrane:

#1

(Drug Eluting Stents):ab,ti,kw OR (Stents, Drug-Eluting):ab,ti,kw OR (Stents, Drug Eluting):ab,ti,kw OR (Drug-Eluting Stent):ab,ti,kw OR (Drug Eluting Stent):ab,ti,kw OR (Stent, Drug-Eluting):ab,ti,kw OR (Drug-Coated Stents):ab,ti,kw OR (Drug Coated Stents):ab,ti,kw OR (Stents, Drug-Coated):ab,ti,kw OR (Stents, Drug Coated):ab,ti,kw OR (Drug-Coated Stent):ab,ti,kw OR (Drug Coated Stent):ab,ti,kw OR (Stent, Drug-Coated):ab,ti,kw

#2

(Drug Eluting Balloons):ab,ti,kw OR (Balloons, Drug-Eluting):ab,ti,kw OR (Balloons, Drug Eluting):ab,ti,kw OR (Drug-Eluting Balloon):ab,ti,kw OR (Drug Eluting Balloon):ab,ti,kw OR (Balloon, Drug-Eluting):ab,ti,kw OR (Drug-Coated Balloons):ab,ti,kw OR (Drug Coated Balloons):ab,ti,kw OR (Balloons, Drug-Coated):ab,ti,kw OR (Balloons, Drug Coated):ab,ti,kw OR (Drug-Coated Balloon):ab,ti,kw OR (Drug Coated Balloon):ab,ti,kw

#3

(De novo coronary lesions ):ab,ti,kw OR (De novo coronary disease):ab,ti,kw OR (de novo coronary stenosis):ab,ti,kw OR (de novo small coronary artery disease):ab,ti,kw OR (de novo small coronary artery lesions):ab,ti,kw OR (de novo coronary bifurcation lesions):ab,ti,kw OR (de novo coronary bifurcation disease):ab,ti,kw OR (Coronary Artery Disease):ab,ti,kw OR (Artery Disease, Coronary):ab,ti,kw

#4

(randomized controlled trial):ab,ti,kw OR (randomized):ab,ti,kw OR (placebo):ab,ti,kw OR (RCT):ab,ti,kw

#5

#1 AND #2 AND #3 AND #4
