# Supplementary material for: Drug-Coated Balloons versus Drug-Eluting Stents for the Treatment of De Novo Coronary Artery Disease: A Meta-Analysis of Randomized Controlled Trials
Source: Rev Cardiovasc Med. 2024 Dec 19;25(12):446. doi: 10.31083/j.rcm2512446 (PMC11683689; doi:10.31083/j.rcm2512446)
Supplement: Supplementary file 1 [file 2153-8174-25-12-446-s1.zip › Supplemental material 2.docx]

Supplemental material 2 Baseline clinical characteristics

| Study | Age | | Male | | DM | | Hyperlipidemia | | Hypertension | |
| --- | --- | --- | --- | --- | --- | --- | --- | --- | --- | --- |
|  | DCB | DES | DCB | DES | DCB | DES | DCB | DES | DCB | DES |
| Latib 2012 BELLO | 64.8±8.5 | 64.8±8.5 | 72 | 71 | 39 | 35 | 71 | 73 | 72 | 75 |
| Cortese 2010 PICCOLETO | 68±9 | 67±10 | 22 | 22 | 13 | 11 | 17 | 13 | 21 | 20 |
| Tang 2018 RESTORE SVD China | 60.1±10.5 | 60.5±10.8 | 77 | 88 | 46 | 48 | 61 | 55 | 78 | 86 |
| Cortese 2020 PICCOLETO II | 66 (50-82) | 64 (48-80) | 83 | 87 | 45 | 40 | 72 | 63 | 77 | 76 |
| Jeger 2020 BASKET-SMALL 2 | 67.18±10.33 | 68·42±10.32 | 295 | 262 | 122 | 130 | 262 | 259 | 324 | 332 |
| Yu 2021 | 62.6±8.8 | 64.0±10.5 | 62 | 56 | 16 | 23 | 52 | 39 | 50 | 54 |
| Gobić 2017 | 56.6 ± 13.2 | 54.3 ± 10.6 | 29 | 27 | 2 | 2 | 4 | 4 | 13 | 13 |

To be continued

| Study | Smoke | | Prior MI | | Prior PCI | | Prior CABG | | Type of DES |
| --- | --- | --- | --- | --- | --- | --- | --- | --- | --- |
|  | DCB | DES | DCB | DES | DCB | DES | DCB | DES |  |
| Latib 2012 BELLO | 15 | 10 | 46 | 33 | 52 | 39 | 9 | 12 | Taxus Libertè |
| Cortese 2010 PICCOLETO | NA | NA | 5 | 6 | 3 | 4 | 3 | 4 | Taxus Libertè |
| Tang 2018 RESTORE SVD China | 34 | 36 | 26 | 28 | 45 | 38 | 0 | 1 | Resolute |
| Cortese 2020 PICCOLETO II | 23 | 19 | 45 | 34 | 59 | 60 | 4 | 4 | XIENCE Prime |
| Jeger 2020 BASKET-SMALL 2 | 226 | 195 | 160 | 133 | 235 | 241 | NA | NA | XIENCE Prime |
| Yu 2021 | 46 | 42 | 18 | 13 | 11 | 14 | 1 | 3 | Resolute |
| Gobić 2017 | 17 | 21 | NA | NA | NA | NA | NA | NA | BioMime |

a. Values are average or n (%).

b. Abbreviations: DM = diabetes mellitus; MI = myocardial infarction; PCI = percutaneous coronary intervention; CABG = coronary artery bypass grafting
