# Supplementary material for: Drug-Coated Balloons versus Drug-Eluting Stents for the Treatment of De Novo Coronary Artery Disease: A Meta-Analysis of Randomized Controlled Trials
Source: Rev Cardiovasc Med. 2024 Dec 19;25(12):446. doi: 10.31083/j.rcm2512446 (PMC11683689; doi:10.31083/j.rcm2512446)

Target lesion revascularization (TLR)


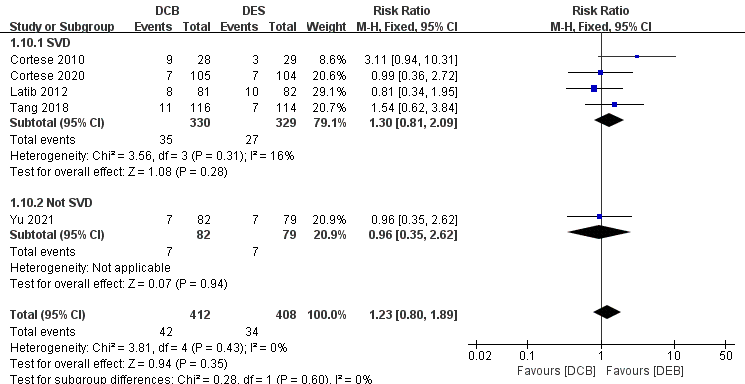


In-lesion late lumen loss (LLL)


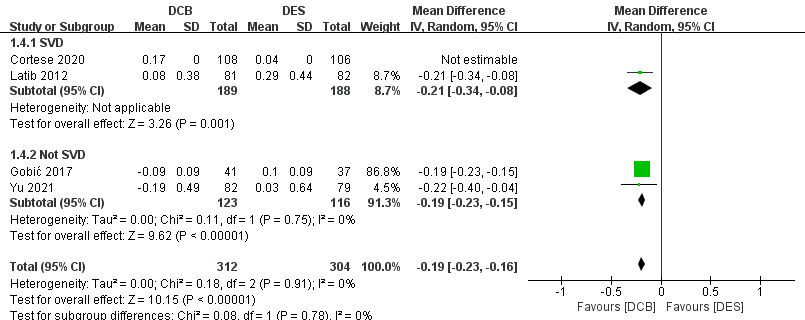


All-cause death


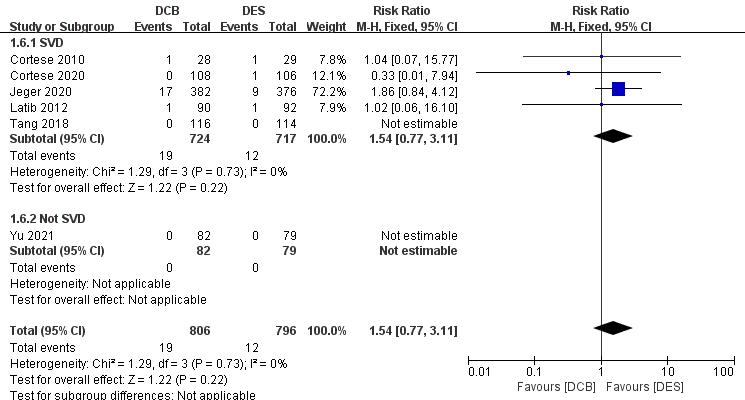


Myocardial infarction (MI)


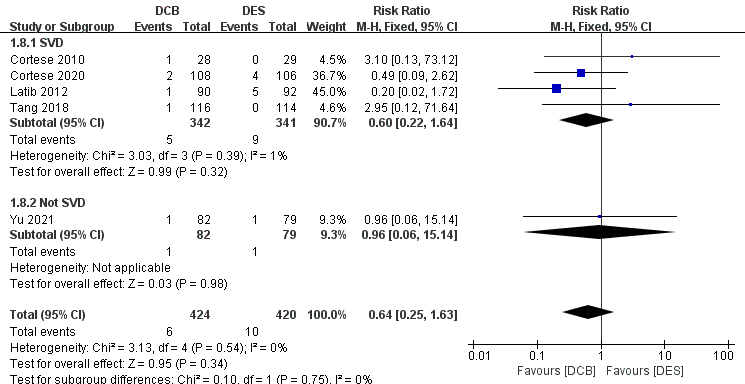


Binary restenosis (BR)


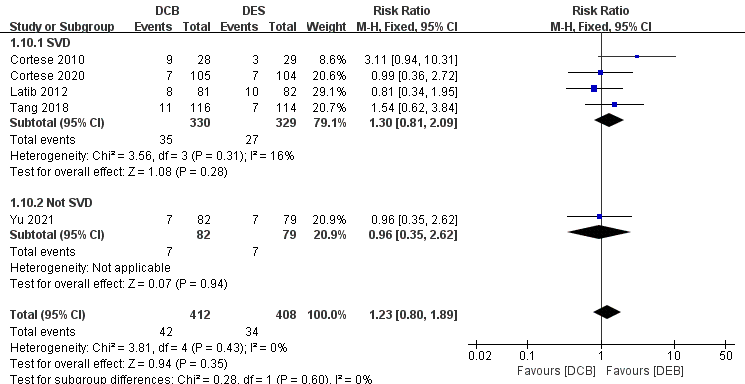

Supplement: Supplementary file 1 [file 2153-8174-25-12-446-s1.zip › Supplemental material 3.docx]
